# Concentrations of Lead, Mercury, Arsenic, Cadmium, Manganese, and Aluminum in the Blood of Pakistani Children with and without Autism Spectrum Disorder and Their Associated Factors

**DOI:** 10.3390/ijerph18168625

**Published:** 2021-08-15

**Authors:** Mohammad H. Rahbar, Shahnaz H. Ibrahim, Syed Iqbal Azam, Manouchehr Hessabi, Fatima Karim, Sori Kim, Jing Zhang, Nasreen Gulzar Ali, Katherine A. Loveland

**Affiliations:** 1 Department of Epidemiology, Human Genetics, and Environmental Sciences, School of Public Health, The University of Texas Health Science Center at Houston, Houston, TX 77030, USA; 2 Division of Clinical and Translational Sciences, Department of Internal Medicine, McGovern Medical School, The University of Texas Health Science Center at Houston, Houston, TX 77030, USA; 3 Biostatistics/Epidemiology/Research Design (BERD) Core, Center for Clinical and Translational Sciences (CCTS), The University of Texas Health Science Center at Houston, Houston, TX 77030, USA; Manouchehr.Hessabi@uth.tmc.edu (M.H.); Sori.Kim@uth.tmc.edu (S.K.); Jing.Zhang.1@uth.tmc.edu (J.Z.); 4 Department of Pediatrics and Child Health, Aga Khan University, Karachi 74800, Pakistan; shahnaz.ibrahim@aku.edu (S.H.I.); nasreen.ali@aku.edu (N.G.A.); 5 Child Development and Rehabilitation Centre, Aga Khan University, Karachi 74800, Pakistan; 6 Department of Community Health Sciences, Aga Khan University, Karachi 74800, Pakistan; iqbal.azam@aku.edu (S.I.A.); fatima.karim1@yahoo.com (F.K.); 7 Department of Biostatistics & Data Science, School of Public Health, The University of Texas Health Science Center at Houston, Houston, TX 77030, USA; 8 Louis A Faillace, MD, Department of Psychiatry and Behavioral Sciences, McGovern Medical School, The University of Texas Health Science Center at Houston, Houston, TX 77054, USA; Katherine.A.Loveland@uth.tmc.edu

**Keywords:** autism spectrum disorder, interaction, GST genes (*GSTP1*, *GSTM1*, *GSTT1*), metals, Pakistan

## Abstract

Background: Autism Spectrum Disorder (ASD) is a neurodevelopmental disorder with early onset in utero or childhood. Environmental exposure to six metals (Pb, Hg, As, Cd, Mn, Al) is believed to be associated with ASD directly or interactively with genes. *Objective*: To assess the association of ASD among Pakistani children with the six metals and genotype frequencies of three GST genes (*GSTP1*, *GSTM1*, *GSTT1*). Methods: We enrolled 30 ASD cases, age 2–12 years old, and 30 age- and sex-matched typically developing (TD) controls in Karachi, Pakistan. We assessed associations of ASD status with various factors using Conditional Logistic Regression models. We also used General Linear Models to assess possible interaction of blood Mn and Pb concentrations with the three GST genes in relation to ASD status. Results: The unadjusted difference between ASD and TD groups in terms of geometric mean blood Pb concentrations was marginally significant (*p* = 0.05), but for Al concentrations, the adjusted difference was marginally significant (*p* = 0.06). Conclusions: This is the first study reporting six blood metal concentrations of Pakistani children with ASD. Estimates provided for possible interactions of GST genes with Mn and Pb in relation to ASD status are valuable for designing future similar studies.

## 1. Introduction

Autism Spectrum Disorder (ASD) is a complex neurodevelopmental disorder that manifests in early childhood. ASD affects language development, communication, imagination, and social interactions. Some features of ASD include repetitive, stereotyped behaviors [1,2]. The prevalence of ASD appears to be on the rise in developed countries [3,4,5,6,7,8]. Public attention to ASD has increased through the 1990s in the US and Europe, and efforts are now underway to close gaps in the existing epidemiologic data [9]. However, reliable epidemiologic data on ASD in developing countries are rare. The prevalence of ASD in Pakistan is unknown, but a school-based study reported a prevalence of 1 in 500 for students with special needs, including ASD; however, this could be severely underestimated because many children with ASD are kept away from attending schools due to social stigma [10]. Additionally, there is no organized effort at the government level, which specifically serves the needs of children with ASD [10,11]. Only private institutions work in this area. On the other hand, some parents may not feel comfortable seeking help for their children with ASD due to the social stigma of ASD [12,13].

In 2007, Rahbar et al. (2010) conducted a survey of 348 general practitioners in Karachi, Pakistan, and reported that only 44% had heard of ASD [14]. Another study in Lahore, Pakistan, demonstrated that although significantly more physicians had reported encountering youth with ASD in their clinical practice, other healthcare providers, such as psychologists and speech therapists, were significantly more likely to correctly identify characteristics associated with an ASD diagnosis according to the DSM-IV-TR [15]. In addition, a study focused on primary school teachers in different districts of Karachi showed that 55% of the teachers have learned about ASD through the media, and only 9% had formal training through workshops related to Autism [16]. Although Bailey et al. [17] and Samms-Vaughan [18] have recognized the global need for ASD research, and some recent epidemiologic studies of the etiology of ASD have been reported from developing countries, no large-scale studies focused on the etiology or prevalence of ASD have been conducted in Pakistan.

The etiology of ASD is believed to be multifactorial and associated with environmental factors [19] either additively or interactively with genes [20]. Exposure to a variety of environmental contaminants has been associated with developmental toxicity in humans [21,22,23,24,25,26,27]. In particular, several studies have investigated the possible association between exposure to environmental toxins (e.g., heavy metals) and ASD [28,29,30,31,32,33,34,35,36,37,38].

The glutathione S-transferase (GST) enzymes play an important role in the cellular detoxification and excretion of environmental pollutants. For example, GST enzymes mediate the detoxification of heavy metals and other xenobiotic compounds by catalyzing the conjugation of glutathione (GSH) to compounds, including xenobiotics. In addition, GST enzymes can conjugate GSH to compounds containing an electrophilic center and thus play an important role in protecting against oxidative stress [39]. Several studies have linked oxidative stress, the imbalance between levels of reactive oxygen species (ROS) and antioxidant levels in the body, with ASD [40]. Levels of GSH, the major cellular antioxidant [41], as well as the ratio of reduced to oxidized GSH, were lower in children with ASD compared to children without ASD [42,43,44], suggesting the involvement of oxidative stress in the disorder. Other studies have linked markers of oxidative stress, such as increased lipid peroxidation [45] and altered vascular characteristics [46], to ASD. Therefore, variants in the genes coding for these GST enzymes may be associated with ASD. 

From our Epidemiological Research on Autism in Jamaica (ERAJ) and ERAJ-Phase 2, we reported a lack of associations between ASD status and each of six metals (lead (Pb), arsenic (As), manganese (Mn), cadmium (Cd), mercury (Hg), and aluminum (Al)) in additive models [47,48,49,50,51,52]. However, our recent findings from Jamaica indicated that ASD status is potentially an effect modifier of the relationship between each of the GST genes (*GSTP1* and *GSTT1*) and some blood heavy metals concentrations. For example, Rahbar et al. reported a significant interaction between *GSTP1* and blood Mn concentrations, indicating that among children who had the Ile/Ile genotype for *GSTP1*, those with Mn ≥ 12 µg/L had about four times higher odds of ASD than those with Mn < 12 µg/L, (*p* = 0.03) [53]. Moreover, they found that the interaction between Mn and *GSTP1* in relation to ASD remained significant with a similar magnitude of association after adjusting for the mixture of four other metals (Pb, Hg, As, and Cd) based on an estimated mixture score using Weighted Quantile Sum (WQS) [54]. While exploring interactive associations between each of the three GST genes and a mixture of the six metals in relation to ASD, Lee et al. developed a generalized WQS (gWQS) regression model that takes into account possible interactions between elements used in a mixture or with other covariates using dependent matched paired data. Using the gWQS method developed, Lee et al. reported the association of a mixture of three metals (Pb, Hg, and Mn) with ASD appeared to differ by *GSTP1* genotype with a marginally significant interaction effect (*p* = 0.07) [55]. Furthermore, they found a significant interaction between Mn (categorized into four quartiles) and *GSTT1* in relation to ASD (*p* = 0.02) [56]. In addition, Rahbar et al. reported a significant interaction between *GSTP1* and ASD status in relation to blood Hg concentrations either in codominant or dominant genetic models for *GSTP1* (*p* < 0.001, *p* = 0.01, respectively) [57]. Interestingly, they also found an interaction between ASD and *GSTP1* in relation to concentrations of Al, As, and Hg in separate models, suggesting that detoxification of these metals may be different between ASD cases and typically developing (TD) controls. Rahbar et al. also reported an interaction between the *GSTP1* and *GSTT1* genes in relation to ASD with a matched odds ratio of 2.97 [58], though they have suggested replication of these results in other populations, where the level of environmental exposure and genetic susceptibility are different from those in Jamaica.

Elevated blood Pb concentrations in Pakistani children are a concern [59] and have been reported in previous studies [60,61,62,63,64]. In addition, some studies reported elevated As [65,66,67], Mn [68], Cd [69], Hg [70,71], and Al [72] levels in children and adults in several communities in Pakistan. Considering that Pakistan has five ethnic groups and is genetically diverse, it is an ideal population for conducting studies focused on genes, environment, and potential interactions among these factors in relation to ASD. 

As the first step in conducting a comprehensive study of the interaction between each of these three GST genes with any of the six metals, we conducted a pilot study to demonstrate the feasibility and obtain the necessary information about the distributions of each of these six metals and the distributions of the genotypes of these GST genes in Pakistani children. We also planned to perform exploratory analyses to estimate various effect sizes that are needed to determine the sample size needed to detect the observed effect sizes with adequate statistical power (e.g., 80%) in future studies.

## 2. Materials and Methods

### 2.1. General Description

In order to extend our autism research in Jamaica (ERAJ) [48,55,73] to other populations living in developing countries with different environments, dietary intake, ethnic background, and ancestries, our team with multidisciplinary researchers led by Dr. Rahbar, who has extensive experience in maternal and child health issues in Pakistan, [14,60,61,74] collaborated with a team at the Aga Khan University (AKU), led by Dr. Shahnaz Ibrahim. Dr. Ibrahim is an experienced pediatric neurologist who has established a database of children with ASD at the AKU child development program. In collaboration with our colleagues at AKU, we conducted a pilot age- and sex-matched case-control study to develop and evaluate the capacity for creating a database of genetic and phenotypic information in Pakistan among ASD cases and TD controls and evaluate the feasibility of shipping biological specimens from Pakistan to the United States for assessment of heavy metals and genetics analyses.

### 2.2. Study Design and Populations of ASD Cases and TD Controls

The Vineland Adaptive Behavior Scales (VABS) (Vineland-3) [75] was administered to parents of a group of Pakistani children between 2–12 years old who visited Pediatrics clinics affiliated with AKU, to evaluate adaptive functioning (daily life skills) with respect to age norms. If the subdomain v-scale (Mean = 15, SD = 3) was under 13, the child was classified as potentially having intellectual and developmental disabilities. For these children (v-scale < 13), the Childhood Autism Rating Scale (CARS) [76] was administered to determine whether the initial clinical criteria for ASD as evaluated by the neurologist were met. Based on the CARS cutoff ≥ 30 and Diagnostic and Statistical Manual of Mental Disorders, Fourth Edition Text Revision (DSM-IV-TR) [77], we identified 30 ASD cases. For each case, we sampled one age- and sex-matched (age ± 6 months) control without developmental disabilities (according to scores on the VABS). As a result, we selected 30 typically developing (TD) controls (children ages from 2 to 12 years) with a subdomain v-scale greater than or equal to 13.

Each parent/guardian was asked to consent to a developmental and behavioral assessment of their child aged 2–12 years, and the child’s assent was also taken in a few typically developing children who were 7–12 years old. Parents also completed a food frequency questionnaire and socioeconomic status (SES) questionnaire.

After the interview, we collected about 4–6 mL of whole blood and 2 mL of saliva from children for assessing exposure to the six heavy metals and frequency of genotypes for the three GST genes (*GSTP1*, *GSTM1*, and *GSTT1*). 

The study protocol was prepared in accordance with the Declaration of Helsinki and approved by the Institutional Review Board (IRB) of the University of Texas Health Science Center at Houston (UTHealth) (HSC-GEN-15-0201) and the Ethical Review Committee (ERC #: 3572-Ped-ERC-15) of AKU. Furthermore, the requirements of the government of Pakistan for human-derived biological samples (HDBS) banks were followed while handling these specimens at UTHealth. The release and/or transfer of the samples was based on the Material Transfer Agreement (MTA) signed by AKU and UTHealth after approval of the grant by the AKU Research Council. 

### 2.3. Sample Processing and Shipment 

The whole blood samples were initially processed by the Infectious Disease Research Lab (IDRL) at the Department of Pediatrics and Child Health for storage at AKU, using two different methods. They were stored in a freezer by maintaining two different temperatures in the same laboratory. First, about 2–3 mL of whole blood was processed for heavy metals and then frozen at −20 °C. The remaining 2–3 mL of blood were processed and stored frozen at −80 °C as a buffy coat and two plasma aliquots within 24 h of collection. All samples were shipped by AKU team to the University of Texas School of Public Health (UTSPH) Human Genetics Center (HGC) in Houston, Texas, USA, for storage during the pilot phase, to assess the feasibility of shipping samples from Pakistan to the US. The UTSPH-HGC assayed 2–3 mL of blood (plasma aliquots) for genotyping *GSTP1, GSTM1,* and *GSTT1* genes and shipped the remaining 2–3 mL of whole blood to the Trace Metals Lab at the Michigan Department of Health and Human Services (MDHHS) in Lansing, Michigan, a Centers for Disease Control and Prevention (CDC)-certified lab, USA, for assessment of the six metal concentrations. Samples were shipped under safe conditions and three different procedures that included (1) the heavy metals samples packed on dry ice, (2) saliva sample at room temperature, (3) buffy coat and two plasma aliquots samples by Cryoport^®^ dry vapor shippers (Irvine, CA, USA).

### 2.4. Assessment of Metal Exposures

The samples shipped to the Trace Metals Lab at MDHHS were assayed for six heavy metals (Pb, Mn, Al, Hg, As, and Cd). The limits of detection (LoD) for Pb, Mn, Al, Hg, As, and Cd were 0.25 μg/dL, 2.5 μg/L, 5.0 μg/L, 0.25 μg/L, 1.3 μg/L, and 0.13 μg/L, respectively. All Pb and Mn concentrations were above the LoD. The percentage of concentrations below LoD for Al, Hg, As, and Cd were 83.3%, 51.7%, 66.7%, and 43.3%, respectively.

### 2.5. Genetic Analysis

Methods for genetic analysis of the *GSTP1* Ile105Val polymorphism (rs1695; C-3217198_20) have been described in detail previously [53,55,57]. Genomic DNA was isolated from the buffy coat using the Gentra PUREGENE Blood Kit (Qiagen, N.V., Venlo, The Netherlands). 

The choice of assays used for the three genes (*GSTP1, GSTM1*, and *GSTT1*) was related to the type of polymorphism that was investigated. The genetic variant in *GSTP1* is a single nucleotide polymorphism (SNP) that results in an amino acid change at position 105 of the encoded protein (Ile105Val). There are three different possible genotypes, and all of them can be distinguished from each other: Ile/Ile, Ile/Val, and Val/Val. We reported the *GSTP1* gene using different genetic models, including the dominant (Val/* vs. Ile/Ile) and the codominant model (Ile/Ile, Ile/Val, and Val/Val), and analyzed *GSTP1* using the dominant model. For the *GSTM1* and *GSTT1* genes, a deletion/insertion polymorphism was evaluated. Since the assay does not distinguish between a normal homozygote (I/I) and a heterozygote (I/D), we considered only a recessive model using a binary variable to represent the genotype: I* and DD. 

### 2.6. Data Management 

For data management, we used Research Electronic Data Capture (REDCap) [78]. All information and data collected for the study were entered into REDCap. We performed double data entry to minimize data entry errors. The first round of data entry was performed by our team in Pakistan. Then the forms were scanned and sent to the US, through an UTHealth secure, shared drive. Since the information in the complete forms was in the Urdu language, we trained a Graduate Research Assistant at UTHealth who was familiar with Urdu to perform the second round of data entry in REDCap. We performed data cleaning and data quality assurance procedures on the data in order to identify missing data as well as data that were out of range or did not comply with the code-book and to resolve the discrepant data before they were analyzed. 

### 2.7. Statistical Analysis 

As part of descriptive analyses, we compared the distribution of demographic characteristics, socioeconomic characteristics, dietary factors, and environmental factors between ASD cases and TD controls using conditional logistic regression (CLR) models. To minimize the influence of measurements below the limits of detection (LoD) and/or skewed distributions of metals in our study, concentrations of each of the six metals that were below the LoD were replaced by the LoD/√2. Since the distribution of blood concentrations of the six metals (Pb, Hg, As, Cd, Mn, and Al) was skewed, we transformed the data using the natural logarithm (ln) in order to produce distributions that better approximated a normal distribution. The means of the log-transformed blood metal concentrations were transformed back to their original scale by applying the natural exponential function, herein called geometric means.

For *GSTT1* and *GSTM1*, as the genotyping assay does not differentiate between a normal homozygote (I/I) and a heterozygote (I/D), we used a recessive model using a binary variable: I/* (I/I or I/D) and DD (null allele). As for the *GSTP1* Ile105Val polymorphism, there are three genotypes (Ile/Ile, Ile/Val, and Val/Val). As the Val/Val level had only three cases for ASD and zero cases for TD control, we assumed a dominant genetic model (Ile/Ile vs. Val/*), and combined Ile/Val and Val/Val levels into one group. 

Univariable General Linear Models (GLMs) with the log-transformed blood metal concentrations were used to identify possible associations between ASD status and the concentrations of the metals for the two metals that have no data below LoD, namely Pb and Mn. For the remaining four metals with data below LoD, Al, As, Hg, and Cd, we have utilized LoD divided by the square root of two. In multivariable GLMs for Pb and Mn, we assessed the relationship between ASD status and the concentrations of metals while controlling for potential confounding variables (based on our previous publications) that included maternal age, paternal education level, and SES (i.e., car ownership by the family) and dietary consumptions. Similarly, in multivariable GLMs for Al, As, Hg, and Cd, we utilized LoD divided by the square root of two. In all GLMs, we controlled for the clustering effect of matching by including an appropriate number of dummy variables that represented the matched pairs (e.g., 29 dummy variables for 30 matched pairs). We fitted GLM models unadjusted and adjusted for potential confounders and reported geometric mean concentrations by ASD status along with *P*-values. 

In multivariable CLR, we also assessed potential interactive associations between each of the GST genes and Pb and Mn concentrations in relation to ASD status. For Pb and Mn, all concentrations were above LoD, so the median (50th quantile) was used as the cutoff (above 50th quantile vs. below 50th quantile). In our previous studies in Jamaica, we used the 75th percentile as the cutoff for Pb and Mn [55], but due to the exploratory nature of this study, the sample size was limited, and thus the median was used as the cutoff.

## 3. Results

At the time of enrollment, the mean ages of ASD cases and TD controls were 78.3 months and 78.5 months, respectively. Eighty percent (80%) of both the ASD cases and TD controls were male. More than half (53.3%) of the ASD cases and TD controls were Urdu speaking. Similarly, 58.3% of mothers and 51.7% of fathers were Urdu speaking. A higher proportion of both the mothers (20.7%) and fathers (41.4%) of ASD cases were age 35 or greater at the time of the child’s birth compared to the mothers (10.7%) and fathers (31.0%) of TD controls. Similarly, the mothers (96.3%) and fathers (96.4%) of ASD cases had higher levels of education than the mothers (71.4%) and fathers (77.8%) of TD controls. ASD cases were of a lower SES compared to TD controls, with 86.7% of case families owning a car versus 93.3% of car ownership by control families. The frequencies of *GSTT1*, *GSTM1*, and *GSTP1* genotypes were not significantly different between ASD cases and TD controls (all *p* > 0.44). The frequencies of characteristics of children and their parents are shown in Table 1.

A comparison of dietary factors between ASD cases and TD controls revealed that a significantly lower proportion of ASD cases reported eating liver/kidney (Matched Odds Ratio (MOR) = 4.33, 95% CI: (1.24, 15.21), *p* = 0.02). ASD cases had significantly lower consumptions of dairy products/eggs, including yogurt (MOR = 5.33, 95% CI: (1.55, 18.30), *p* = 0.01), eggs (MOR = 8.00, 95% CI: (1.00, 63.96), *p* = 0.05) and cheese (MOR = 6.50, 95% CI: (1.47, 28.80), *p* = 0.01). Root vegetables, such as carrot, pumpkin (MOR = 4.67, 95% CI: (1.34, 16.24), *p* = 0.02) and yam or sweet potato (MOR = 10.00, 95% CI: (1.28, 78.12), *p* = 0.03) showed significantly lower proportion in ASD cases as well. Among the leafy vegetables, cauliflower and broccoli revealed a significantly lower proportion of ASD cases (MOR = 9.00, 95% CI: (1.14, 71.04), *p* = 0.04). In addition, compared to ASD controls, ASD cases consumed significantly lower servings of almost all fruits (all *p* ≤ 0.05, except figs (*p* = 0.05), banana (*p* = 0.14) and other melon (*p* = 0.10)). Moreover, a comparison of environmental factors between ASD cases and TD controls revealed that a significantly lower proportion of ASD cases reported drinking piped water.

There were dietary differences between ASD cases and TD cases that showed a marginal significance, which may be due to the smaller sample size. The proportion of ASD cases in regard to the consumption of river fish (MOR = 2.75, 95% CI: (0.88, 8.64), *p* = 0.08), and canned food (MOR = 4.50, 95% CI: (0.97, 20.83), *p* = 0.05) was marginally significant compared to TD controls. The frequency distributions of eating other types of food in ASD and TD children are shown in Table 2.

Our results from the unadjusted GLM, reported in Table 3, showed a marginally significant higher geometric mean blood lead (Pb) concentration for TD controls in comparison to ASD cases (7.68 μg/dL vs. 6.37 μg/dL; *p* = 0.05). In the GLM, after adjusting for maternal age, paternal education level, and SES, we did not find a significant association between blood lead concentrations and ASD status (7.11 μg/dL for ASD cases vs. 8.48 μg/dL for TD controls; *p* = 0.16). While the difference between the unadjusted geometric mean of blood aluminum (Al) concentrations between ASD cases and TD was not significant (4.05 μg/L for ASD cases vs. for TD controls 3.92 μg/L; *p* = 0.68), the adjusted mean difference was marginally significant (4.49 μg/L for ASD cases vs. 3.69 μg/L for TD controls; *p* = 0.06), after adjusting for maternal age, paternal education level, SES, and consumption of root vegetables (yam, sweet potato, or dasheen). In addition, the univariable unadjusted GLMs also showed no significant differences between geometric mean blood metal concentrations of ASD cases and TD controls for arsenic (1.15 μg/L vs. 1.12 μg/L; *p* = 0.74), mercury (0.29 μg/L vs. 0.29 μg/L; *p* = 0.96), cadmium (0.14 μg/L vs. 0.15 μg/L; *p* = 0.84), and manganese (13.97 μg/L vs. 13.93 μg/L; *p* = 0.97). In the GLMs, for the aforementioned four metals after adjusting for maternal age, paternal education level, SES, and metal-specific dietary consumption of the child, we did not find a significant difference between geometric mean blood metal concentrations of the ASD cases and TD controls (all *p* > 0.30). 

Table 4 displays the unadjusted and adjusted associations between metals and *GSTT1* genotypes in relation to ASD status based on interactive CLR models. We did not find a significant interaction between blood metal concentration and *GSTT1* genotypes. However, there was a meaningful interaction between Manganese (Mn) and *GSTT1* genotype (DD) (Unadjusted MOR = 0.22, 95% CI: (0.01, 4.01), *p* = 0.31, *p*-value for interaction = 0.31; Adjusted MOR = 0.16, 95% CI: (0.01, 3.46), *p* = 0.24, *p*-value for interaction = 0.27). *p*-value for the MOR for blood Pb concentration and *GSTT1* genotypes (I*) was meaningful for both adjusted (*p* = 0.31) and unadjusted (*p* = 0.24) model. 

Multivariable CLR was used to investigate the interaction of blood Pb and Mn concentrations with *GSTP1* genotypes in relation to ASD (Table 5). In addition to the variables in the unadjusted model, we adjusted for SES. Due to the limited sample size, we used the dominant genetic model (Val/* vs. Ile/Ile) instead of a codominant model (Val/Val vs. Ile/Val vs. Ile/Ile) for *GSTP1* genotypes. Although in the model displayed in Table 5 we did not find a statistically significant interaction between blood metal concentrations and *GSTP1* genotypes, we estimated the MOR based on an interaction between manganese (Mn) and *GSTP1* genotype (Val/*) (Unadjusted MOR = 0.57, 95% CI: (0.11, 3.06), *p* = 0.51, *p*-value for interaction = 0.40). 

As shown in Table 6, we investigated the interaction of each metal (Pb and Mn) concentration with *GSTM1* genotypes in relation to ASD. Although there were no significant interactions detected, the *p*-values to test MOR for blood Pb concentration at *GSTM1* DD displayed meaningful magnitude and direction of the associations (Unadjusted MOR = 0.48, 95% CI: (0.10, 2.21), *p* = 0.34; Adjusted MOR = 0.43, 95% CI: (0.09, 2.11), *p* = 0.30). 

## 4. Discussion

### 4.1. Association of Blood Metal Concentrations in Relation to ASD 

The main hypothesis in our ASD study in Pakistan involves comparisons of the blood concentrations of each of the six metals between ASD cases and TD controls. As shown in Table 3, in univariable GLMs Pb was the only metal that had a marginally significant lower blood concentration in ASD cases compared to that of TD controls (*p* = 0.05). However, after adjusting for potential confounding variables, maternal age, father’s education level, and socioeconomic status, this difference was no longer statistically significant at a 5% level of significance, (*p* = 0.16). In addition, the difference in the geometric mean of blood aluminum concentration between ASD cases and TD controls was marginally significant after adjusting for maternal age, father’s education level, socioeconomic status, and consumption of root vegetables (yam, sweet potato, or dasheen) (*p* = 0.06). For the other metals, including Hg, the difference in blood metal concentrations between ASD cases and TD control groups were not significant in either univariable or multivariable GLMs that adjusted for the aforementioned confounding variables. These findings are consistent with our findings from our ERAJ study in Jamaica [47,48,49,50,51,52]. Although there was no conclusive evidence of additive associations between exposure to each metal and ASD status, the unadjusted and adjusted geometric mean of blood metal concentrations for both ASD cases and TD controls in Pakistani children provide a valuable reference for future studies in similar populations.

### 4.2. Association of GST Genes in Relation to ASD 

In this study, we reported the genotype distribution of the three GST genes (*GSTP1, GSTM1*, and *GSTT1*) in Pakistani children with and without ASD. Reporting of such frequency distributions of GST genotypes is the first for children in Pakistan and one of the very few in child populations with and without ASD in South Asian countries. 

For example, the distribution of *GSTP1* in Pakistan showed a lower proportion of Val/Val genotype (for children with ASD: 10.0%, for TD controls: 0.0%), which differs from findings from our Jamaican study at the *GSTP1* Val/Val genotype (for children with ASD 19.4%, for TD controls 23.3%) [57]. However, a study conducted in Lagos, Nigeria with children (4–14 years old) with ASD and age-matched TD cases showed findings closer to the Pakistani study, reporting 19.0% of ASD cases and 4.4% of TD cases with the *GSTP1* Val/Val genotype [79]. In contrast, the frequency of *GSTP1* Ile/Ile genotype was higher in the Pakistani study (ASD 46.7%, TD 56.7%) than that in the Jamaican study (ASD 25.9%, TD 24.4%), and the Nigerian study (ASD 38.1%, TD 47.8%) [79]. *GSTM1* in our study in Pakistan displayed different distributions from those in studies from different countries. In our study, the percentage of *GSTM1* DD genotype in the ASD cases and TD controls were 50.0% and 40.0%, respectively, whereas, for the same genotype, the proportions were 29.7% and 23.6% in the Jamaican study [57]. This also differs from the finding from the Nigerian study, where it was reported that 33.3% of ASD cases and 13.0% of TD cases had the *GSTM1* DD genotype [79]. A case-control study in a youth population (2–18 years old) in Semerang and Solo, Indonesia with 51 ASD cases 45 unrelated TD controls reported that 11.8% of ASD cases and 6.7% of TD controls had the *GSTM1* DD genotype [80]. The proportion of ASD cases and TD controls with *GSTT1* DD genotype in our Pakistani study was 17.2% and 13.3%, respectively, while in the Jamaican study, it was 26.6% and 24.8%; [57] in the Nigerian study, 11.9% and 4.3% [79], and in the Indonesian study, 39.2% and 31.1% [80]. The differences in the frequency of the three GST genes in our Pakistani population relative to other populations support the need for additional studies in the region.

Furthermore, the distribution of genotypes of GST genes between ASD cases and TD control groups can serve as a valuable reference for study designs with a larger sample size that involves additive or interactive associations of GST genes with ASD status in the same or similar populations. Our comparisons of the distribution of genotypes of GST genes between ASD cases and TD control groups revealed no significant differences (all *p* ≥ 0.44). This finding is in line with findings from a study with children (3–16 years old) with ASD cases (*n* = 90) and age- and sex-matched TD controls (*n* = 76) conducted in Pakistani cities (Islamabad, Khanewal, and Lahore), where *GSTT1* (unadjusted OR (95% CI): 1.81 (0.82, 4.03); *p* = 0.14) and *GSTM1* (unadjusted OR (95% CI): 1.23 (0.66, 2.29); *p* = 0.51) showed similar results [81]. However, our reported relative frequency or Matched Odds Ratio (MOR) of the genotypes for these three GST genes can be used to design future studies that involve the assessment of the effects of GST genes and environmental exposure to the six heavy metals. 

### 4.3. Interactive Association of GST Genes and Blood Concentrations of Heavy Metals in Relation to ASD 

Although our study in Pakistan was not initially geared towards investigating the interactive effects of GST genes and blood concentration of six heavy metals, findings regarding the magnitude of the effect size assessed through the MORs provide relevant information to design such studies in the future. For example, the unadjusted and adjusted geometric mean of blood metal concentrations and the frequency of genotypes for the three GST genes (*GSTP1, GSTM1*, and *GSTT1*) in Pakistani children with and without ASD reported from this pilot study can be used to design new studies to determine the interactive association between GST genes and blood metal concentration in relation to ASD among children in Pakistan or other countries in the South Asian subcontinent that have similar genotype distributions for the three GST genes.

Due to the limited sample size of this pilot study, we believe there was not sufficient statistical power to detect a significant interaction between Mn and *GSTT1* genotype (DD) in relation to ASD status. However, the magnitude and the direction of the association (Unadjusted MOR = 0.22, 95% CI: (0.01, 4.01), *p* = 0.31, *p*-value for interaction = 0.31; Adjusted MOR = 0.16, 95% CI: (0.01, 3.46), *p* = 0.24, *p*-value for interaction = 0.27) are in line with our previous reported findings from the ERAJ study that showed a significant interaction between *GSTT1* genotypes and blood metals concentrations (BMC) in relation to ASD by Rahbar et al. (Range 3 (2nd quartile ≤ Mn < 3rd quartile) vs. Range 1 (Mn < 1st quartile): Unadjusted MOR = 0.52, 95% CI: (0.20, 1.38), *p* = 0.19, *p*-value for interaction = 0.02; Range 3 vs. Range 1: Adjusted MOR = 0.58, 95% CI: (0.21, 1.58), *p* = 0.29, *p*-value for interaction = 0.01) [56]. Similarly, the magnitude and the direction of the association in Pakistani children (Unadjusted MOR = 0.48, 95% CI: (0.15, 1.55), *p* = 0.22; Adjusted MOR = 0.41, 95% CI: (0.12, 1.40), *p* = 0.15) was in agreement with the findings from the Jamaican study (Range 2 (1st quartile ≤ Pb < 2nd quartile) vs. Range 1 (Pb < 1st quartile): Unadjusted MOR = 0.51, 95% CI: (0.28, 0.93), *p* = 0.03; Range 3 vs. Range 1: Unadjusted MOR = 0.41, 95% CI: (0.22, 0.73), *p* < 0.01; Range 4 (Pb ≥ 4th quartile) vs. Range 1 (Pb < 1st quartile): Unadjusted MOR = 0.42, 95% CI: (0.22, 0.78), *p* < 0.01) [55] regarding the MOR for blood Pb concentration and *GSTT1* genotypes (I*) in relation to ASD.

The magnitude and the direction of the association between Mn and *GSTP1* genotype (Val/*) (Unadjusted MOR = 0.57, 95% CI: (0.11, 3.06), *p* = 0.51, *p*-value for interaction = 0.40) in our Pakistani study was higher than the findings from our Jamaican study, where significant interaction between blood Mn concentration and *GSTP1* in relation to ASD is observed (Unadjusted MOR = 0.77, 95% CI: (0.47, 1.25), *p* = 0.29, *p*-value for interaction = 0.03) [55]. This is because reciprocal of the reported unadjusted MOR (1/0.57) in our Pakistani study was 1.75, whereas the reciprocal of the unadjusted MOR (1/0.77) in our Jamaican study was 1.30. The unadjusted MOR and 95% CI for blood Pb concentrations at *GSTP1* Ile/Ile were 0.48 (0.10, 2.41) with *p* = 0.38 for unadjusted model, which was in agreement but with lower reciprocal of MOR (1/0.48 = 2.08) than the those (Range 2 (1st quartile ≤ Pb < 2nd quartile): vs. Range 1 (Pb < 1st quartile): 1/0.48 = 2.08; Range 3 (2nd quartile ≤ Pb < 3rd quartile) vs. Range 1 (Pb < 1st quartile): 1/0.25 = 4.00; Range 4 vs. Range 1: 1/0.25 = 4.00) of the results in Rahbar et al. (2020) [55], where the unadjusted MOR and 95% CI is 0.48 (0.16, 1.50), 0.25 (0.09, 0.76), and 0.25 (0.08, 0.77), for Range 2 vs. Range 1, Range 3 vs. Range 1, Range 4 vs. Range 1, respectively. *p*-values for the MORs were *p* = 0.21, *p* < 0.01, and *p* < 0.01. These examples demonstrate the utility of findings from this pilot study in future investigations on the association of GST genes and blood concentrations of heavy metals in relation to ASD in similar populations.

In Rahbar et al. (2020) [55], the unadjusted MORs and their 95% CIs for blood Pb concentrations at *GSTM1* DD were 0.30 (0.11, 0.84) with *p* = 0.02 for Range 3 vs. Range 1, and 0.29 (0.11, 0.75) with *p* < 0.01 for the Range 4 vs. Range 1. However, in this study the unadjusted MOR blood Pb concentration at *GSTM1* DD displayed findings that were not significant in the unadjusted model, (Unadjusted MOR = 0.48, 95% CI: (0.10, 2.21), *p* = 0.34). This difference could be partly explained by a much larger sample size in the Jamaican study than this study. 

### 4.4. Blood Concentrations of Metals in Pakistani Children with and without ASD 

Although reports of the blood concentrations of some of these six metals, such as Pb, are scarce in the literature for Pakistani children under 12 years old, [61,62,64,82,83] some of the findings regarding blood concentrations of metals, such as Al, Cd, or Hg, are reported for the first time. New reports on such blood metal concentrations in Pakistani children with ASD serve as a valuable resource to further investigate this population or other populations in South or Southeast Asia. The new information reported on TD controls regarding blood metal concentrations could serve as a valuable reference for further research on Pakistani children, as well as being useful to public health officials in future studies and potential interventions.

Blood concentrations of Pb and Mn were found to be higher in our Pakistani TD controls than Jamaican TD controls with age 2–8 years, respectively (Pb: 7.68 μg/dL vs. 2.34 μg/dL; Mn: 13.93 μg/L vs. 10.30 μg/L). Blood concentrations of As and Cd were similar for TD controls in both populations (As: 1.19 μg/L vs. 2.29 μg/L; Cd: 0.15 μg/L vs. 0.20 μg/L). Blood concentrations of Hg and Al were reported to be lower in our Pakistani TD controls than Jamaican TD controls (Hg: 0.32 μg/L vs. 0.81 μg/L; Al: 4.02 μg/L vs. 9.74 μg/L) [55]. These differences in blood metal concentrations between the two populations may be due to various dietary, environmental, genetic, or socioeconomic factors, which will be subject to further investigation.

## 5. Limitations 

We acknowledge that blood may not be the most suitable biomarker for assessing exposures to all six metals reported here. For example, hair specimens are better than blood for assessment of Hg [84] and Mn [85]. Urine is considered a better biomarker for the assessment of exposure to As (and speciation) [86] and Cd [87]. However, blood is considered a good biomarker for the assessment of Pb [88] and Al [89].

We also acknowledge some other limitations in this study. Although we used Conditional Logistic Regression and LoD cutoffs to minimize the potential bias in the estimation of geometric mean blood concentrations of metals, such as Al, As, Hg, and Cd, we understand that substituting blood concentrations below LoD by LoD/√2 may still cause some bias in the estimation of means, as well as their respective standard deviations. The bias may also be caused by the percentage below LoD for Al, As, Hg, and Cd (43.3–83.3%) and the overall limited size of the sample (i.e., 30 matched pairs: 60 children).

We also acknowledge that the limited sample size of this pilot study (*n* = 60; 30 pairs) may not have provided adequate power, resulting in marginal or non-significant associations found in this pilot study, particularly the interactive associations between exposure to each metal and ASD status. However, our limited sample size had a lesser impact on the findings in effect size estimates provided for assessing interactions between each of the metals and the GST genotypes; hence, these estimates could be useful in designing future studies. Additional studies with greater sample sizes can clarify the interactions between each GST genotype and the blood metal concentrations of Pakistani children with and without ASD, enabling comparison with the findings from similar populations in different regions or countries.

## 6. Conclusions

Although there was no conclusive evidence of any additive associations between exposure to each metal and ASD status, the geometric means of blood metal concentrations for ASD cases and TD controls from the univariable and multivariable GLMs provide useful information on children with and without ASD from Pakistan. In addition, reporting on the genotype distribution of the three GST genes (*GSTP1, GSTM1,* and *GSTT1*) in Pakistani children with and without ASD is the first in the population and one of the few reported from studies in Pakistani children with and without ASD in the South Asian subcontinent. The distribution of GST genes differed from the findings in other studies of children with and without ASD in other regions. Similarly, to our knowledge, we are one of the first to report the effect size and direction of association between each of the two heavy metals (Pb and Mn) and GST genes in relation to ASD status in children from Pakistan. These findings are in agreement with previous literature, including the potential interaction between each of the two heavy metals (Mn, Pb) and *GSTP1* or *GSTT1* genes in relation to ASD status. Our findings in relation to additive or interactive associations of heavy metal and GST genes in relation to ASD serve as a reference for future epidemiologic studies in this population or other similar populations to uncover further possible additive or interactive associations between heavy metals, GST genes, and ASD status.

## Figures and Tables

**Table 1:** Characteristics of children and their parents by ASD case status (30 matched pairs).

Variables	Categories	ASD Case N (%)	TD Control N (%)	Matched OR (95% CI)	*p-*Value	*p*-Value ^c^
**Child’s sex**	Male	24 (80.0)	24 (80.0)	1.00 (0.28, 3.54)	1.00	1.00
**Child’s age (months)**	Age < 72	13 (43.3)	13(43.3)	1.00 (0.06, 15.99)	1.00	1.00
	Age ≥ 72	17 (56.7)	17 (56.7)			
**Child’s ethnicity**	Sindhi and Saraeki	7 (23.3)	6 (20.0)	2.24 (0.32, 15.64)	0.61	0.86
	Punjabi	5 (16.7)	4 (13.3)	2.37 (0.30, 18.60)	0.58	
	Urdu speaking	16 (53.3)	16 (53.3)	1.87 (0.33, 10.59)	0.90	
	Other	2 (6.67)	4 (13.3)			
**Maternal age**	<35 years	23 (79.3)	25 (89.3)	2.00 (0.50, 8.00)	0.33	0.33
**(at child’s birth)**	≥35 years	6 (20.7)	3 (10.7)			
**Paternal age**	<35 years	17 (58.6)	20 (69.0)	1.60 (0.52, 4.89)	0.41	0.41
**(at child’s birth)**	≥35 years	12 (41.4)	9 (31.0)			
**Maternal ethnicity**	Sindhi and Saraeki	4 (13.3)	5 (16.7)	1.05 (0.16, 6.96)	0.87	0.88
	Punjabi	4 (13.3)	5 (16.7)	1.01 (0.17, 6.11)	0.80	
	Urdu speaking	19 (63.3)	16 (53.3)	1.64 (0.33, 8.17)	0.41	
	Other	3 (10.0)	4 (13.3)			
**Paternal ethnicity**	Sindhi and Saraeki	4 (13.3)	7 (23.3)	1.22 (0.16, 9.41)	0.53	0.48
	Punjabi	5 (16.7)	4 (13.3)	2.48 (0.33, 18.89)	0.51	
	Urdu speaking	18 (60.0)	13 (43.3)	2.96 (0.54, 16.31)	0.20	
	Other	3 (10.0)	6 (20.0)			
**Maternal** **education ^a^**	Up to high school	1 (3.7)	8 (28.6)	10.4 (1.20, 90.09)	0.02	0.02
**(at child’s birth)**	Beyond high school	26 (96.3)	20 (71.4)			
**Paternal** **education ^a^**	Up to high school	1 (3.6)	6 (22.2)	7.71 (0.86, 69.10)	0.05	0.05
**(at child’s birth)**	Beyond high school	27 (96.4)	21 (77.8)			
**Parental** **education**	Both up to high school	0 (0.0)	5 (17.9)	NR ^b^	0.99	0.99 *
**(at child’s birth)**	At least one beyond high school	28 (100.0)	23 (82.1)			
**Socioeconomic status (SES)**	Car ownership	26 (86.7)	28 (93.3)	0.50 (0.09, 2.73)	0.42	0.42
**Home live in**	Owned	23 (76.7)	21 (70)	1.50 (0.42, 5.32)	0.53	0.53
***GSTT1*^de^**	I *	24 (82.8)	26 (86.7)	0.80 (0.22,2.98)	0.74	0.74
	DD	5 (17.2)	4 (13.3)			
***GSTM1*^d^**	I *	15 (50.0)	18 (60.0)	0.70 (0.27,1.84)	0.47	0.47
	DD	15 (50.0)	12 (40.0)			
***GSTP1*** **(codominant)**	Ile/Ile	14 (46.7)	17 (56.7)	NR ^b^	1.00	0.87 *
	Ile/Val	13 (43.3)	13 (43.3)		1.00	
	Val/Val	3 (10.0)	0 (0.0)			
***GSTP1*** **(dominant)**	Ile/Ile	14 (46.7)	17 (56.7)	0.67 (0.24, 1.87)	0.44	0.44
	Val/*	16 (53.3)	13 (43.3)			

^a^ Calculated with Fisher’s exact test due to limited sample size in at least one of the cells. ^b^ NR = Not reported due to unstable estimates caused by having a zero cell in at least one of the cells. ^c^ The *p*-values were based on Wald’s test in conditional logistic regression models that compares the distribution of independent variables between ASD case and TD control groups. ^d^ I/I or I/D indicate the homozygote (I/I) or a heterozygote (I/D) for *GSTT1* and *GSTM1*. ^e^ *GSTT1* was missing for 1 ASD case. * *p-*values may be affected by the cell with the frequency of zero.

**Table 2:** Comparison of dietary factors between ASD case and TD controls using Conditional Logistic Regression (CLR) based on 60 children (30 matched pairs).

Exposure Variables	Category	ASD Case N (%)	TD Control N (%)	MOR (95% CI) *	*p-*Value
**Source of** **drinking water**	Piped water	4 (13.3)	13 (43.4)	0.18 (0.04, 0.82)	0.03
**Seafood**	Lake/Pond fish (catfish, crappie)	7 (23.3)	12 (40.0)	2.50 (0.69, 7.36)	0.18
	Bay fish (speckled trout, redfish, flounder)	11 (36.7)	8 (26.7)	0.57 (0.17, 1.95)	0.37
	River fish (bass, trout)	7 (23.3)	14 (46.7)	2.75 (0.88, 8.64)	0.08
	Offshore fish (tuna, snapper, whiting)	5 (16.7))	5 (16.7)	1.00 (0.25, 4.00)	1.00
	Shellfish (lobster, crab, crawfish)	3 (10.0)	3 (10.0)	1.00 (0.14, 7.10)	1.00
**Meat/Organ**	Beef as main dish	18 (60.0)	23 (76.7)	2.25 (0.69, 7.31)	0.18
	Lamb as main dish	2 (6.7)	3 (10.0)	1.50 (0.25, 8.98)	0.66
	Goat as main dish	24 (80.0)	25 (83.3)	1.25 (0.34, 4.66)	0.74
	Chicken as main dish	30 (100.0)	28 (93.3)	NR **	1.00
	Liver, kidney	4 (13.3)	14 (46.7)	4.33 (1.24, 15.21)	0.02
**Dairy** **products/eggs**	Milk	25 (83.3)	29 (96.7)	5.80 (0.63, 53.01) *	0.20
	Yogurt	11 (36.7)	24 (80.0)	5.33 (1.55, 18.30)	0.01
	Eggs	22 (73.3)	29 (96.7)	8.00 (1.00, 63.96)	0.05
	Cheese	8 (26.7)	19 (63.3)	6.50 (1.47, 28.80)	0.01
**Root vegetables**	Carrot, pumpkin	12 (40.0)	23 (76.7)	4.67 (1.34, 16.24)	0.02
	Yam, sweet potato	2 (6.7)	11 (36.7)	10.00 (1.28, 78.12)	0.03
**Leafy vegetables**	Lettuce	10 (33.3)	10 (33.3)	1.00 (0.38, 2.66)	1.00
	Cauliflower, broccoli	9 (30.0)	17 (56.7)	9.00 (1.14, 71.04)	0.04
	Cabbage	10 (33.3)	15 (50.0)	2.67 (0.71, 10.05)	0.15
	Turnip	4 (13.3)	10 (33.3)	3.00 (0.81, 11.08)	0.10
	Spinach	16 (53.3)	15 (50.0)	0.86 (0.29, 2.55)	0.78
**Fruits**	Oranges	14 (46.7)	26 (86.7)	13.00 (1.70, 99.38)	0.01
	Tangerine	7 (23.3)	22 (73.3)	8.50 (1.96, 36.79)	<0.01
	Grapes	12 (40.0)	26 (86.7)	15.00 (1.98, 113.56)	<0.01
	Apples	15 (50.0)	27 (90.0)	13.00 (1.70, 99.38)	0.01
	Pineapples	2 (6.7)	9 (30.0)	8.00 (1.00, 63.96)	0.05
	Figs	1 (3.3)	7 (23.3)	8.82 (1.01, 76.96) *	0.05
	Peach	6 (20.0)	20 (66.7)	5.67 (1.66, 19.34)	<0.01
	Plums	3 (10.0)	17 (56.7)	15.00 (1.98, 113.56)	<0.01
	Strawberry	6 (20.0)	23 (76.7)	9.50 (2.21, 40.79)	<0.01
	Blackberry	1 (3.3)	8 (26.7)	8.00 (1.00, 63.96)	0.05
	Banana	22 (73.3)	26 (86.7)	5.00 (0.58, 42.80)	0.14
	Watermelon	10 (33.3)	22 (73.3)	7.00 (1.59, 30.80)	0.01
	Other melon (cantaloupe, honeydew)	10 (33.3)	17 (56.7)	2.40 (0.85, 6.81)	0.10
**Other food related questions**	Canned food	6 (20.9)	13 (43.3)	4.50 (0.97, 20.83)	0.05
	Aluminum foil	1 (3.3)	1 (3.3)	1.00 (0.06, 15.99)	1.00
	Unpeeled fruits	7 (23.3)	19 (63.3)	13.00 (1.70, 99.38)	0.01
	Animal fat	2 (6.7)	0 (0.0)	NR **	1.00

* Calculated with Fisher’s exact test due to limited sample size in at least one of the cells. ** NR = Not reported due to unstable estimates caused by having a zero cell in at least one of the cells.

**Table 3:** Geometric mean of blood metal concentrations based on univariable and multivariable General Linear Models (GLMs) that account for four possible confounders and potential clustering effects of the matched pairs of ASD cases and TD controls (30 matched pairs or 60 children).

Metal	Limits of Detection (LoD)	% Below LoD	Geometric Mean ^a^ (Based on Univariable GLMs)				Adjusted Geometric Mean ^a^ (Based on Multivariable Adjusted GLMs) *			
			ASD Cases	TD Controls	Mean Difference ^b^	*p* Value ^c^	ASD Cases	TD Controls	Mean Difference ^b^	*p* Value ^c^
**Al ^d^**	5.0 μg/L	83.3%	4.05	3.92	0.13	0.68	4.49	3.69	0.80	0.06
**As ^e^**	1.3 μg/L	66.7%	1.15	1.12	0.03	0.74	1.47	1.29	0.18	0.30
**Hg ^f^**	0.25 μg/L	51.7%	0.29	0.29	–0.00	0.96	0.24	0.20	0.05	0.40
**Cd ^g^**	0.13 μg/L	43.3%	0.14	0.15	–0.00	0.84	0.16	0.16	–0.00	0.88
**Pb ^h^**	0.25 μg/dL	0.0%	6.37	7.68	–1.32	0.05	7.11	8.48	–1.37	0.16
**Mn ^i^**	2.5 μg/L	0.0%	13.97	13.93	0.04	0.97	12.75	12.25	0.50	0.70

^a^ We reported geometric mean (Exp. (Mean (ln metal concentrations))); For calculation of means we used “**Least Square (LS) means**”. ^b^ Mean differences indicate the difference between mean blood metal concentrations in ASD cases and TD controls. ^c^ The *P*-values in this table are for testing H0: Exp. (Mean (ln metal concentrations for the ASD group)) = Exp. (Mean (ln metal concentrations for the TD control group)) using GLMs. ^d^ For Al, LoD cutoff line was used, and factors adjusted for included: maternal age, paternal education level, SES, and consumption of root vegetables (yam, sweet potato, or dasheen). ^e^ For As, the LoD cutoff line was used, and factors adjusted for included: maternal age, paternal education level, SES, and consumption of cabbage. ^f^ For Hg, the LoD cutoff line was used, and factors adjusted for included: maternal age, paternal education level, SES, and frequency of seafood consumption. ^g^ For Cd, the LoD cutoff line was used, and factors adjusted for included: maternal age, paternal education level, SES, and consumption of root vegetables (yam, sweet potato, or dasheen). ^h^ For Pb, factors adjusted for included: maternal age, paternal education level, and SES. ^i^ For Mn, factors adjusted for included: maternal age, paternal education level, SES, and consumption of offshore fish. * For adjusted models, there was one missing value in maternal age for ASD cases, two missing values in maternal age for TD controls, two missing values in paternal education level for ASD cases, and three missing values in paternal education level for TD controls.

**Table 4:** Potential interaction of *GSTT1* genotypes with blood Lead (Pb), Manganese (Mn) concentration in relation to ASD in Pakistani children based on CLR model. (N = 30 matched pairs or 60 children).

Metal	Binary	*GSTT1 * Genotype (s) ^a^	Unadjusted ^b^			Adjusted ^c^		
			Matched OR (95% CI)	*p* Value ^d^	*p*-Value for GSTT1*Metal Interaction	Matched OR (95% CI)	*p* Value ^d^	*p-*Value for GSTT1*Metal Interaction
**Pb**	>50th vs. ≤50th	I *	0.48 (0.15, 1.55)	0.22	0.99	0.41 (0.12, 1.40)	0.15	0.99
	>50th vs. ≤50th	DD	NR	NR		NR	NR	
**Mn**	>50th vs. ≤50th	I *	1.18 (0.30, 4.63)	0.81	0.31	1.04 (0.26, 4.24)	0.95	0.27
	>50th vs. ≤50th	DD	0.22 (0.01, 4.01)	0.31		0.16 (0.01, 3.46)	0.24	

NR = Not reported due to unstable estimates caused by a frequency of zero in at least one of the cells. ^a^ *GSTT1* was missing for 1 ASD case. ^b^ **CLR unadjusted Model 1:** logit *p* (ASD = 1) = β1 (Metal > cutoff) + β2 (*GSTT1 DD*) + β3 (Metal > cutoff *GSTT1 DD). ^c^ Adjusted for SES. ^d^ *p-*values were based on Wald’s test in conditional logistic regression models.

**Table 5:** Interaction of *GSTP1* genotypes with blood Lead (Pb), Manganese (Mn) concentration (under a dominant genetic model) in relation to ASD in Pakistani children based on CLR model. (N = 30 matched pairs).

Metal	Binary	*GSTP1* Genotype (s) ^a^	Unadjusted ^b^			Adjusted ^c^		
			Matched OR (95% CI)	*p* Value ^d^	*p* Value for GSTP1*Metal Interaction	Matched OR (95% CI)	*p* Value ^d^	*p* Value for GSTP1*Metal Interaction
**Pb**	>50th vs. ≤50th	Val/*	0.69 (0.16, 2.99)	0.62	0.74	0.67 (0.15, 2.93)	0.59	0.73
	>50th vs. ≤50th	Ile/Ile	0.48 (0.10, 2.41)	0.38		0.46 (0.09, 2.35)	0.35	
**Mn**	>50th vs. ≤50th	Val/*	0.57 (0.11, 3.06)	0.51	0.40	0.56 (0.10, 3.06)	0.50	0.53
	>50th vs. ≤50th	Ile/Ile	1.40 (0.29, 6.74)	0.68		1.12 (0.22, 5.88)	0.89	

^a^*GSTP1* Val/* = Ile/Val or Val/Val. ^b^ **CLR unadjusted Model 2:** logit *p* (ASD = 1) = β1 (Metal > cutoff) + β2 (GSTP1 Val/*) + β3 (Metal > indicator function for the metal being above cutoff *GSTP1 Val/*). ^c^ Adjusted for SES. ^c^ *p-*values were based on Wald’s test in conditional logistic regression models.

**Table 6:** Interaction of *GSTM1* genotypes with blood Lead (Pb), Manganese (Mn) concentration (under a dominant genetic model) in relation to ASD in Pakistani children based on the CLR model. (N = 30 matched pairs).

Metal	Binary	*GSTM1* Genotype (s) ^a^	Unadjusted ^a^			Adjusted ^b^		
			Matched OR (95% CI)	*p-*Value ^c^	*p-*Value for GSTM1*Metal Interaction	Matched OR (95% CI)	*p-*Value ^c^	*p-*Value for GSTM1*Metal Interaction
**Pb**	>50th vs. ≤50th	I *	0.65 (0.16, 2.69)	0.56	0.75	0.62 (0.15, 2.61)	0.51	0.73
	>50th vs. ≤50th	DD	0.48 (0.10, 2.21)	0.34		0.43 (0.09, 2.11)	0.30	
**Mn**	>50th vs. ≤50th	I *	0.71 (0.15, 3.28)	0.66	0.69	0.56 (0.11, 2.87)	0.49	0.62
	>50th vs. ≤50th	DD	1.04 (0.23, 4.72)	0.96		0.93 (0.20, 4.36)	0.92	

^a^**CLR unadjusted Model 1:** logit *p* (ASD = 1) = β1 (Metal > cutoff) + β2 (*GSTM1 DD*) + β3 (Metal > cutoff * *GSTM1 DD*). ^b^ *A*djusted for SES. ^c^ *p-*values were based on Wald’s test in conditional logistic regression models.

## Data Availability

The data from this study are not publicly available due to the requirements of the collaborators in this study. However, after five years from the time that this initial manuscript is published, data presented in this study could become available upon request from the corresponding author based on the following: (1) a commitment to using the data only for research purposes and not to identify any individual participant; (2) a commitment to using best statistical and ethical practices in analyzing and reporting finding; (3) a commitment to securing the data using appropriate information technology; (4) a commitment to crediting the source and the funding agencies of the original project in all publications and presentations, and (5) a commitment to destroying or returning the data after analyses are completed.
